# Genetic structure of four socio-culturally diversified caste populations of southwest India and their affinity with related Indian and global groups

**DOI:** 10.1186/1471-2156-5-23

**Published:** 2004-08-19

**Authors:** Revathi Rajkumar, VK Kashyap

**Affiliations:** 1 DNA Typing Unit, Central Forensic Science Laboratory, 30 Gorachand Road, Kolkata, India-700014

## Abstract

**Background:**

A large number of microsatellites have been extensively used to comprehend the genetic diversity of different global groups. This paper entails polymorphism at 15 STR in four predominant and endogamous populations representing Karnataka, located on the southwest coast of India. The populations residing in this region are believed to have received gene flow from south Indian populations and world migrants, hence, we carried out a detailed study on populations inhabiting this region to understand their genetic structure, diversity related to geography and linguistic affiliation and relatedness to other Indian and global migrant populations.

**Results:**

Various statistical analyses were performed on the microsatellite data to accomplish the objectives of the paper. The heretozygosity was moderately high and similar across the loci, with low average G_ST _value. Iyengar and Lyngayat were placed above the regression line in the R-matrix analysis as opposed to the Gowda and Muslim. AMOVA indicated that majority of variation was confined to individuals within a population, with geographic grouping demonstrating lesser genetic differentiation as compared to linguistic clustering. D_A _distances show the genetic affinity among the southern populations, with Iyengar, Lyngayat and Vanniyar displaying some affinity with northern Brahmins and global migrant groups from East Asia and Europe.

**Conclusion:**

The microsatellite study divulges a common ancestry for the four diverse populations of Karnataka, with the overall genetic differentiation among them being largely confined to intra-population variation. The practice of consanguineous marriages might have attributed to the relatively lower gene flow displayed by Gowda and Muslim as compared to Iyengar and Lyngayat. The various statistical analyses strongly suggest that the studied populations could not be differentiated on the basis of caste or spatial location, although, linguistic affinity was reflected among the southern populations, distinguishing them from the northern groups. Our study also indicates a heterogeneous origin for Lyngayat and Iyengar owing to their genetic proximity with southern populations and northern Brahmins. The high-ranking communities, in particular, Iyengar, Lyngayat, Vanniyar and northern Brahmins might have experienced genetic admixture from East Asian and European ethnic groups.

## Background

The Indian subcontinent is regarded as a natural genetic laboratory, owing to the co-existence and interaction of socio-culturally, linguistically, ethnically and genetically diversified endogamous populations in a geographical terrain. It is believed that the earliest humans leaving Africa for Eurasia might have taken a coastal route across Saudi Arabia, through Iraq, Iran, to Pakistan and finally entered India along the coastlines [1]. A second wave of migration (~10,000 years ago) brought in Proto-Dravidian Neolithic farmers from Afghanistan, who were later displaced southwards by a large influx of Indo-European speakers ~3500 years ago in to the subcontinent [2,3]. The origin and settlement of the Indian people still remains intriguing, fascinating scientists to explore the impact of these past and modern migrations on the genetic diversity and structure of contemporary populations [4-6].

Anthropologically, southern and northern populations are distinct and these differences are further substantiated by (i) the presence of Neolithic sites in this region suggests that Neolithic people of southern India came from north by land and the west-coast by sea [7], (ii) the southern megaliths resemble closely with those of the Mediterranean and western-Europe, while those from northern India are similar to megaliths found in Iran and Baluchisthan [8], and (iii) the predominance of Dravidian language in this region as opposed to their secluded occurrence in central Asia and other parts of India, suggests that the Dravidian languages might have originated within India [9]. It is, thus, of considerable genetic interest to understand the genetic structuring and relationships of southern populations.

The present study was carried out on one of the largest southern states, Karnataka, positioned on the southwest coast of India, with a dwelling of about 50 million people. This expanse has been a rich source of prehistoric discoveries dating back to the Paleolithic era that are akin to those seen in Europe [7]. Karnataka has received continuous gene flow from different caste and linguistic groups residing in the adjoining areas of Maharashtra, Andhra Pradesh and Tamil Nadu [10], resulting in the congregation of a large number of diverse endogamous groups within this region. Its large coastline of about 400 Km also attracted the Portugese, Dutch and French traders, who were seeking more profitable ventures on the southern coast at large [2]. Southwest India is, thus, one of the most disparate terrains, with extensive colonization in the past and justifies an in-depth genetic study.

A few studies utilizing classical markers have been carried out on southern populations [5,11,12], including few communities of Karnataka [13,14]. However, sound inferences relating to their genetic structuring and diversity could not be drawn due to low discriminatory power of these markers. Recently, microsatellite markers have gained immense popularity in precisely defining population structure, diversity, affinities, gene flow and other crucial aspects associated with population genetics [15-21] because of the relative expediency, with which a large number of loci and alleles can be typed, facilitating the accumulation of vast data sets that can be readily analyzed with an extensive array of statistical tools [22,23]. These markers also demonstrate high heterozygosity [24], rendering them highly suitable for carrying out the present study.

Among the different caste and tribal groups inhabiting the southwest coast of India, we have selected four predominant Dravidian-speaking communities from Karnataka: Iyengar Brahmin, Lyngayat, Gowda and Muslim, they not only belong to dissimilar groups of the Indian caste hierarchy but also have varied migration histories, conferring them uniqueness and significance from a genetic perspective. The present microsatellite study primarily attempts to understand the genetic structure of the four selected populations and to determine their genetic relationship with other linguistically and ethnically similar groups of southern India and Brahmin groups of northern India. It has been suggested that that despite the linguistic homogeneity in southern India, these populations have remained genetically diversified [25]. Hence, we sought to determine the role played by geographical location and linguistic affiliation in genetically differentiating Indian populations. Also, as mentioned earlier, the western coast has witnessed colonization from different world populations, we aim to divulge the impact of these past migrations on the gene pool of the present southern populations by discerning their relationship with historically acclaimed and established migrant groups, ethnically represented by European, Hispanic, East Asian and African populations.

## Results

**Figure 1:**
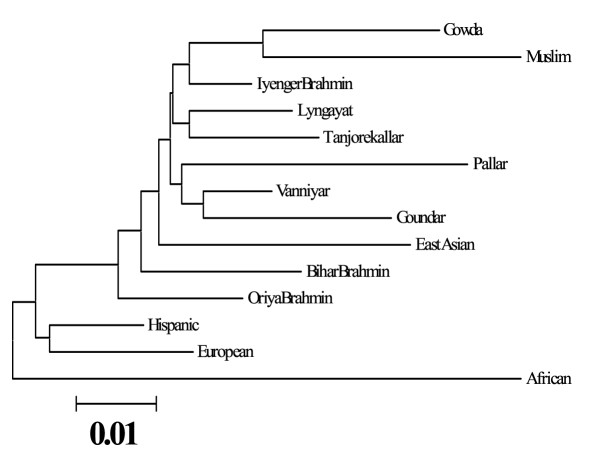
Neighbor-joining tree depicting the genetic relationship of Karnataka populations with related Indian and global ethnic groups based on 15 STR markers.

Allele frequency at 15 STR was used to compute the heterozygosity (observed) for the four studied populations, which varied for each locus, and population but reflected similar values, ranging between 0.724 and 0.797 (Table 1). An average G_ST _value of 0.009 elucidates the low degree of genetic differentiation in them. However, the G_ST _value for the pooled Indian and global populations demonstrated a high value at 2.3% (data not shown). Genetic relationship of studied populations with other similar southern groups; Vanniyar, Gounder, Pallar and Tanjore Kallar [26,27], northern Brahmins belonging to Orissa [28] and Bihar [29], and four relevant global ethnic groups: European, Hispanic, African [30] and East Asian [31] was divulged by computing DA distances (Table 2) and represented using NJ tree (Fig. 1). Among the four studied populations, Iyengar, Gowda and Muslim formed a distinct cluster. Although NJ tree clearly depicts the clustering of southern populations, D_A _distances indicate that among these groups, Iyengar, Lyngayat and Vanniyar are more similar to the northern Brahmins (0.030). Furthermore, genetic distances emphasize the affinity of Lyngayat with Tanjore Kallar (0.029), Iyengar (0.026) and Vanniyar (0.028). Estimation of relatedness between the southern and global populations shows that all the southern communities formed a separate cluster, nevertheless, genetic distances disclose the affinity of upper caste Indian communities; Iyengar, Lyngayat, Vanniyar, Bihar and Oriya Brahmin with Europeans and East Asians. The Indian populations were most distant to Africans.

**Table 1:** Average heterozygosity and G_ST _values for 15 loci in the four studied populations.

	OBSERVED HETEROZYGOSITY				
		
LOCUS					G_ST_
	BRAHMIN	LINGAYAT	GOWDA	MUSLIM	
TPOX	0.707	0.581	0.542	0.555	0.010
D3S1358	0.661	0.793	0.559	0.488	0.036
THO1	0.815	0.785	0.678	0.688	0.005
D21S11	0.876	0.857	0.779	0.733	0.005
D18S51	0.907	0.938	0.779	0.888	0.006
PENTA E	0.921	0.876	0.864	0.800	0.014
D5S818	0.692	0.724	0.525	0.733	0.007
D13S317	0.753	0.714	0.745	0.733	0.007
D7S820	0.723	0.734	0.754	0.800	0.005
D16S539	0.861	0.846	0.830	0.777	0.010
CSF1PO	0.723	0.734	0.745	0.733	0.009
PENTA D	0.815	0.755	0.741	0.933	0.006
vWA	0.784	0.734	0.779	0.688	0.002
D8S1179	0.861	0.822	0.745	0.755	0.007
FGA	0.861	0.894	0.803	0.911	0.017
Average	0.797	0.785	0.724	0.747	0.009

**Table 2:** D_A _distance matrix between ten Indian and four global groups based on allele frequency at 15 microsatellites.

**Pop**	**HS**	**NE**	**MO**	**CA**	**OB**	**IB**	**LY**	**GO**	**MU**	**BB**	**PL**	**VN**	**TK**	**GD**
**HS**														
**NE**	0.079													
**MO**	0.049	0.123												
**CA**	0.029	0.086	0.07											
**OB**	0.044	0.092	0.052	0.04										
**IB**	0.044	0.091	0.041	0.04	0.03									
**LY**	0.047	0.096	0.047	0.04	0.03	0.026								
**GO**	0.066	0.122	0.072	0.07	0.055	0.036	0.047							
**MU**	0.076	0.118	0.078	0.07	0.066	0.051	0.056	0.054						
**BB**	0.043	0.101	0.052	0.05	0.038	0.031	0.037	0.054	0.068					
**PL**	0.061	0.11	0.067	0.07	0.064	0.05	0.056	0.075	0.077	0.063				
**VN**	0.045	0.105	0.042	0.04	0.034	0.023	0.028	0.039	0.053	0.037	0.049			
**TK**	0.047	0.096	0.053	0.05	0.044	0.028	0.029	0.052	0.062	0.044	0.052	0.032		
**GD**	0.064	0.112	0.064	0.06	0.051	0.036	0.043	0.057	0.073	0.054	0.059	0.032	0.043	

**Abbreviations used in Table- **Hispanic – HS, African – AF, Asian – AS, European – EU, OriyaBrahmin – OB, Iyengar Brahmin – IB, Lyngayat – LY, Gowda – GO, Muslim – MU, Bihar Brahmin – BB, Pallar – PL, Vanniyar – VN, Tanjore Kallar – TK, Goundar – GD.

**Figure 2:**
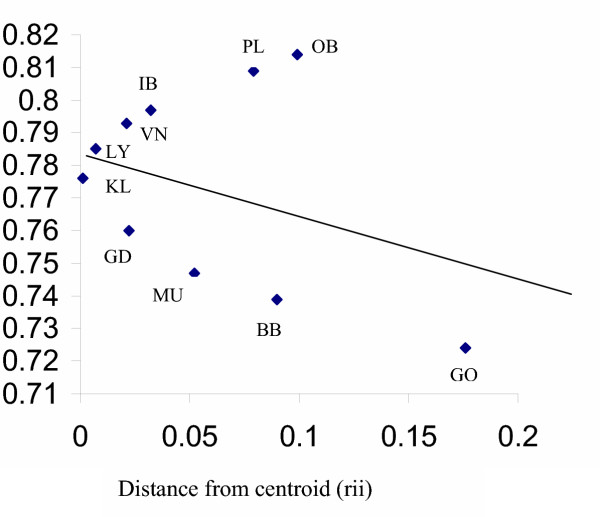
Regression plot demonstrating the relatively higher gene flow levels in high-ranking populations of India. **Abbreviations used in figure: **OB-Oriya Brahmin, PL-Pallar, IB-Iyngar Brahmin, VN-Vanniyar, LY-Lyngayat, TK-Tanjorekallar, GD-Goundar, Mu-Muslim, BB-Bihar Brahmin, GO-Gowda.

The regression model (Fig. 2), of mean per locus heterozygosity against distance from centroid assumes that when a population experiences same amount of gene flow from a homogenous source, a linear relationship exists between the expected and observed heterozygosity. A change in gene flow directly affects this linear relationship. The R-matrix when applied to the Indian populations assists in understanding the influence of external gene flow and admixture among populations. The higher observed than expected heterozygosity of Iyengar and Lyngayat, placed above the theoretical regression line helps infer that these populations have received more than average external gene flow, which was also observed in Vanniyar, Pallar and Oriya Brahmin. The Gowda and Muslim groups exhibit lower than expected heterozygosity values and fall below the regression line, suggesting lesser admixture in them.

The microsatellite diversity computed using AMOVA revealed that the genetic variation observed in Indian populations was mainly confined to variation amongst individuals (~98%), irrespective of their geographic or linguistic grouping (Table 3). The geographical clustering of populations into three regions: north, southwest (Karnataka) and southeast (Tamil Nadu) demonstrated a low variance of 0.29%, p = 0.010 (Table 3a). As compared to geographical grouping, the linguistic clustering (Indo-Caucasian and Dravidian) exhibited a noticeable increase in the molecular variance between the two groups, 0.65% (p = 0.06, Table 3b). The genetic diversity among populations within each group remained almost similar at both levels of analysis.

**Table 3:** Genetic differentiation of Indian populations based on AMOVA

**(a) Geographical grouping**		
Groups in set 1	Source of Variation	Percentage Variation

Group 1 – North: Bihar and Orissa populations	Among groups	0.29
Group 2 – South-west: Karnataka populations	Among populations in groups	0.97
Group 3 – South-east: Tamil Nadu Populations	Within populations	98.74
**(b) Linguistic grouping**		

Groups in set 2	Source of Variation	Percentage Variation
Group 1 – Indo-European: Orissa and Bihar	Among groups	0.69
Group 2 – Dravidian: Southern populations	Among populations in groups	0.94
	Within populations	98.40

## Discussion

In recent years, population genetics has witnessed extensive use of microsatellite markers to understand and evolutionary histories of contemporary human populations [17,32-34]. Though, the populations inhabiting south India have played a major role in formation of the Indian gene pool, however, very few genetic studies have been carried out on them. The present study utilizes 15 STRs to provide comprehensive genetic information on four predominant communities inhabiting the southwest coast of India, which may significantly help in understanding the genetic composition of southern populations.

### Genetic structure of Karnataka populations

The most distinctive feature revealed by the fifteen microsatellites was the considerable genetic homogeneity amongst the four diverse caste groups residing in southwest India. The presence of an almost similar allele frequency pattern [34], suggests that these populations might have a common ancestry or probably experienced very high gene flow during the period of their coexistence. The above finding is further supported by the low genetic differentiation of 1.0% among the studied groups irrespective of their caste and migration histories. The high heterozygosity and rii values in Lyngayat reflect the admixture and stochastic processes experienced by it. The genetic affinity of Lyngayat with other related southern caste populations, like, Iyengar, Vanniyar and Tanjore Kallar reiterates its heterogeneous past. It is noteworthy that although the southern populations exhibited higher affinity amongst each other, the high-ranking populations, like, Iyengar, Lyngayat and Vanniyar also displayed some genetic similarity to Brahmins from Bihar and Orissa, indicating that the gene pool of Iyengar and Lyngayat probably consists of genetic inputs from both southern and northern groups. However, strong conclusions cannot be drawn due to low genetic differentiation among the studied populations. Though the Gowda is known to have moved in to Karnataka from the adjoining area of Tamil Nadu, our study reveals that Gowda cluster with the studied populations and not with Tamil groups. The low hetetozygosity and high rii values of Gowda implies that it might have differentiated as a result of stochastic processes. Furthermore, the relatively lower heterozygosity and admixture levels of Gowda and Muslim might be attributed to the socio-cultural practice of consanguineous marriages in them. The Muslim group was found to be genetically similar to local populations. Regional conversions from diverse castes that occurred during the period of Islamic dominance might elucidate the more or less identical genetic relationship between Muslims and other studied groups. The microsatellite study emphasizes the genetic similarity among the Karnataka populations, with the lack of any strong caste or religious bias in them.

### Analysis of genetic variance

AMOVA test strongly suggests that genetic diversity among the southern populations was mainly confined to intra-population variation, further emphasizing the genetic homogeneity in them. Analysis using different genetic markers corroborate with our finding that the genetic diversity in human populations can be mainly attributed to variation within populations [4,17,19,34,36,37].

An exploration of the genetic differentiation based on geographical grouping of populations discloses the genetic similarity among populations residing in a region. Nevertheless, the geographic affinity was comparably lesser to that observed within the two linguistic families, viz., Dravidian and Indo-European. Our finding provides evidence to the strong linguistic affinity prevailing amongst the Dravidian speaking populations and imparts them genetic distinctness from the Indo-European linguistic group. Even though prior studies have indicated that genetic clusters often correspond closely to predefined regional and linguistic groups [34], AMOVA suggests that caste system along with geographical contiguity are not ideal platforms for differentiating the analyzed Indian populations. It must, however, be acknowledged that use of less number of polymorphisms in this study might plausibly have led to the greater influence of linguistic affiliation on these populations rather than geographical proximity.

### Genetic affinity with global populations

The genetic differentiation of the studied populations with relevant global migrant groups was estimated to be 2.3%, relatively lower than the 9% observed in another similar study [16], which had used a different set of microsatellite markers. Sampling from a confined area, as well as the use of lesser number of loci might have contributed to this apparent difference in the results. The southern populations formed a separate cluster from the world populations. Molecular studies on Indian populations using diverse markers (nuclear, mtDNA and Y-chromosome) have demonstrated that the upper caste populations have higher semblance with Europeans than Asians [26]. Intriguingly, in the present study, communities belonging to the upper strata of the Hindu caste hierarchy, i.e., Iyengar, Lyngayat, Vanniyar and northern Brahmins, displayed almost identical genetic affinity with both Europeans and East Asians. Therefore, all though it is believed that south India remained isolated and cushioned from the foreign invasions, the southern populations, especially, the high-ranking groups might have genetically admixed with migrant groups that entered via the west coast and north. Further exploration of their relationship is essential before drawing concrete conclusions. A more comprehensive picture would emerge on analysis of mtDNA and Y chromosome markers.

## Methods

### The populations

The populations selected in this study comprise of three major Hindu castes-Iyengar, Lyngayat, Gowda and a Muslim community, inhabiting the southwest coastal terrain of Karnataka (11.3 – 18.45°N latitudes and 74.12 – 78.40°E longitudes). All the populations belong to the Dravidian linguistic family and are speakers of the local dialect, Kannada, but differ in caste hierarchy and socio-religious practices. Consanguineous marriages have been reported in Karnataka, with inbreeding levels of the order 0.020 to 0.033, in general [38].

Iyengar hold a high position in the Indian caste hierarchy and sporadic accounts on Brahmin, suggests that they primarily migrated from the upper Gangetic plains to southern India. Nonetheless, few bioanthropological studies have revealed that morphologically Brahmins of a geographical region are similar to the local groups.

Lyngayat community was initially formed, as a religious cult by the amalgamation of people from different castes and geographical regions but later developed into a distinct community practicing strict marriage endogamy with social sub-divisions such as clans, sub-castes and sects [10].

Gowda is a low ranking agriculturist caste group that typically exhibits the Dravidian socio-cultural characteristic of consanguineous marriage. It is believed to have moved in from the adjoining area of Tamil Nadu.

Muslim is a linguistically heterogeneous, complex religio-ethnic group, [10]. It is believed that the invasion of Turks, Afghans (A.D 998–1030) and Moghals during the 15^th ^century, introduced new genes only in northern India, suggesting that Muslims from Southern India are mainly local converts [3].

### Micosatellite loci studied

The 15 STR marker set analyzed in this study consists of thirteen tetra nucleotide repeat loci: D3S1358, THO1, D21S11, D18S51, D5S818, D13S317, D7S820, D16S539, CSF1PO, vWA, D8S179, TPOX, FGA and two penta nucleotide repeat loci: Penta D, Penta E. Their repeat size makes them less prone to slippage of polymerase during enzymatic amplification compared to the dinucleotide repeats, allowing unambiguous typing [20]. The 15 selected loci are situated on 13 different chromosomes, with D5S818 and CSF1PO being present on chromosome 5 and Penta D and D21S11, located on chromosome 21. The alleles across the loci are substantially unlinked, making them suitable for analyzing inter and intra-population genetic diversity.

### STR Typing

The blood samples were collected from unrelated individuals belonging to – Iyengar (65), Lyngayat (98), Gowda (59) and Muslim (45) communities, residing in different districts of Karnataka. DNA was extracted from blood by the phenol-chloroform method [40], followed by quantitation using the QuantiBlot™ kit (Perkin-Elmer, Foster City, CA, USA). Two nanogram of the isolated DNA was used as template for the PCR amplification of the 15 STRs using the PowerPlex™16 kit (Promega Corp., Wisconsin Madison, USA). Raw data were collected with the GeneScan™ software, Ver. 3.2.1 (Applied Biosystems, Foster City, CA, USA) and typed using the PowerTyper™ 16 Macro (Promega Corp., Wisconsin Madison, USA).

### Statistical Analysis

Allele frequencies of the 15 STR loci were calculated using the gene counting method [40]. The genetic diversity (G_ST_), observed heterozygosity and pairwise genetic distances (DA) were computed using allele frequencies [42]. The DA distance is least affected by sample size and can precisely obtain correct phylogenetic trees under various evolutionary conditions [43]. Neighbor-joining trees were constructed using DA distances [44], and its robustness was established by bootstrap resampling procedures.

Analysis of molecular variance (AMOVA) was performed using the Arlequin Ver. 2.00 package [45]. Two levels of analysis were performed to explore the microsatellite diversity among the four studied populations along with six other socio-culturally similar groups inhabiting different regions of India. At the first level, three geographical groups were constructed: (1) north (2) southwest: Karnataka and, (3) southeast: Tamil Nadu, to estimate the genetic variance among populations from diverse geographical regions. The second set of analysis was aimed at investigating the genetic diversity between the Dravidian and Indo-European linguistic family.

To assess the gene flow experienced by these populations, the rii value, i.e., the genetic distance of a population from the centroid was calculated using the regression model [46]. This model utilizes the heterozygosity of each population and the distance from the centroid as the arithmetic mean of allele frequencies:



where, r_ii _is the distance from the centroid, p_i _is the frequency of the allele in i^th ^population and  is the mean allelic frequency.

## List of abbreviations

STR – Short Tandem Repeat

AMOVA – Analysis of Molecular Variance

NJ tree – Neighbor-Joining tree

## Authors' contributions

RR carried out the molecular studies, analyzed the genetic data and drafted the manuscript. VKK participated in the design, conceiving and preparation of manuscript. Both authors read and approved the final manuscript.
